# Impact of High Risk of Obstructive Sleep Apnea on Health-Related Quality of Life: The Korean National Health and Nutrition Survey 2019–2021

**DOI:** 10.3390/jcm13154360

**Published:** 2024-07-25

**Authors:** Min-Seok Chang, Sunmin Park, Jihye Lim, Ji-Ho Lee

**Affiliations:** 1 Department of Internal Medicine, Yonsei University Wonju College of Medicine, Wonju 26426, Republic of Korea; mim8776@yonsei.ac.kr (M.-S.C.); sunmin@yonsei.ac.kr (S.P.); 2 Department of Medical Informatics and Biostatistics, Yonsei University Wonju College of Medicine, Wonju 26426, Republic of Korea; jihye8082@yonsei.ac.kr

**Keywords:** obstructive sleep apnea, quality of life, comorbidities

## Abstract

**Background/Objectives**: Obstructive sleep apnea (OSA) impairs quality of life (QoL). However, its disease burden in the general population remains unknown. We aimed to investigate the association between OSA and health-related QoL in the general Korean population. **Methods**: This study analyzed cross-sectional datasets of adults (≥40 years) in the Korean National Health and Nutrition Examination Survey 2019–2021. QoL was assessed using the 3-level EuroQoL 5-dimension component (EQ-5D-3L). The high risk of OSA was determined using the STOP-Bang questionnaire (score ≥ 3). Demographic and clinical factors were included in linear regression analyses to identify the factors associated with EQ-5D-3L. **Results**: Of the 8966 total participants, 6792 (75.8%) and 2174 (24.2%) were classified as having a low risk and high risk of OSA, respectively. The high risk OSA group showed significantly lower QoL scores when compared with the low risk OSA group (0.939 ± 0.003 vs. 0.951 ± 0.002, *p* < 0.001). However, the mean difference was within the minimal clinically important difference (MCID) of EQ-5D-3L. Only females exceeded the MCID for the EQ-5D-3L. Elderly females with a high risk of OSA showed the lowest QoL. The regression coefficient of high risk OSA in the multivariate model was −0.018 (95% CI: −0.025–−0.01, *p* < 0.001). Patient demographics and comorbidities also showed significant associations with the EQ-5D-3L. Their regression coefficient was higher than that of high risk OSA. **Conclusions**: The impact of high risk OSA on QoL manifested differently according to age and sex. The impact of comorbidities on QoL was greater than that of high risk OSA, highlighting the important role of comorbidities and the need for their adjustment in the assessment of QoL.

## 1. Introduction

Obstructive sleep apnea (OSA) is a common disorder that interrupts breathing repeatedly during sleep by partially or completely blocking the upper airway [1]. The clinical presentation of OSA includes nocturnal symptoms such as loud snoring, observed apneas, and restlessness, as well as daytime symptoms like excessive daytime sleepiness and mood disturbances [2,3]. It is a significant public health problem, affecting a large proportion of the population [4]. The recurrent apneas and hypopneas during sleep not only disrupt the quality of sleep but also lead to medical conditions, from acute diseases to long-term diseases such as stroke, cardiovascular disease, metabolic syndrome, and neuropsychiatric problems causing cognitive dysfunction [5,6].

Many studies have investigated the relationship between OSA and various medical complications. For example, OSA is closely associated with hypertension and cardiovascular disease due to recurrent episodes of apnea-induced intermittent hypoxia. This intermittent hypoxia triggers autonomic, hemodynamic, and biochemical changes, leading to an increased mean arterial pressure and cerebral vascular resistance, which contribute to the pathophysiology of these conditions [7,8]. However, the effect of OSA on quality of life (QoL) remains relatively unrecognized. Considering the impact of sleep disturbance on daily life, the possibility of the deterioration of QoL is quite evident.

Several studies have reported lower QoL in individuals with OSA when compared to those without OSA across physical, mental, and emotional domains [9]. Most studies were conducted on a limited number of participants using a cross-sectional study design and generic QoL questionnaires [10]. However, studies exploring the relationship between OSA and QoL in the general population according to age and sex are scarce. Sex differences in OSA affect the clinical manifestation, with women being more likely to report symptoms like insomnia and fatigue rather than the classic symptoms of loud snoring and witnessed apneas [11]. These differences in symptoms can affect quality of life and the approach to diagnosis and treatment. The prevalence of OSA increases with age. This age-related increase in OSA prevalence can be attributed to various factors, including changes in body composition, upper airway anatomy, and muscle tone. As people age, the impact of OSA on quality of life can become more pronounced, affecting daily functioning and overall health [12]. This study aimed to investigate the relationship between OSA and QoL and to evaluate the differences across various age and sex groups using the Korean National Health and Nutrition Examination Survey (KNHANES) data.

## 2. Materials and Methods

### 2.1. Study Population

**Figure 1:**
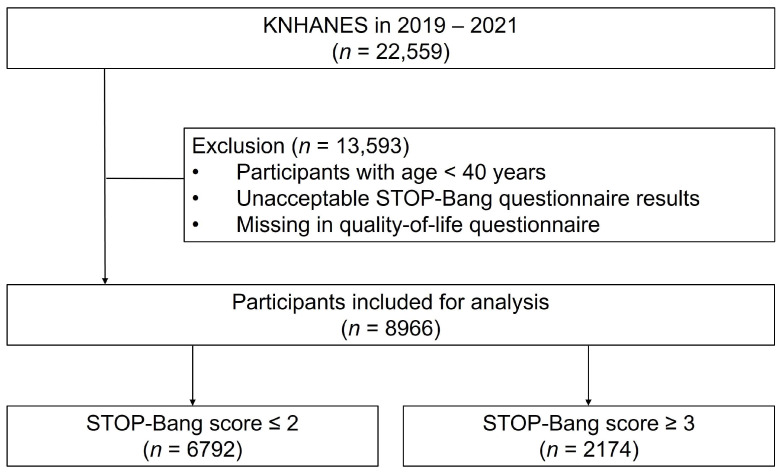
Flowchart of subject selection.

This study utilized data from the eighth KNHANES, conducted between 2019 and 2021. The KNHANES, managed by the Korean Centers for Disease Control and Prevention (KCDC) since 1998, uses probability sampling to assess the health and nutritional status of the noninstitutionalized civilian population in Korea. The survey includes a health interview, a health examination, and a nutrition survey, all conducted by skilled interviewers and laboratory technicians. Detailed information about the KNHANES is available online (https://knhanes.kdca.go.kr/knhanes/eng/index.do, accessed on 30 January 2024). The OSA screening questionnaire was administered only to adults aged 40 years and older. Out of 22,529 participants, 13,593 individuals under 40 years old or with missing data were excluded, resulting in 8966 participants in the study (Figure 1). The study was approved by the KCDC’s Institutional Review Board, and all participants provided informed consent.

### 2.2. Data Collection

Demographic and socioeconomic data, such as age, sex, height, body weight, and household income, were collected. Comorbidities, including asthma, chronic obstructive pulmonary disease (COPD), allergic rhinitis, hypertension, dyslipidemia, stroke, myocardial infarction or angina, diabetes, chronic kidney disease, and depression, were identified based on physician diagnoses. Body mass index (BMI) was calculated by dividing body weight by height squared (kg/m^2^) and was categorized into quartiles as follows: below 18.5 kg/m^2^, 18.5–24.9 kg/m^2^, 25.0–29.9 kg/m^2^, and above 30.0 kg/m^2^. The smoking status was categorized as “never” smokers (never smoked or smoked fewer than 100 cigarettes), “former” smokers (quit for at least 6 months after smoking more than 100 cigarettes), and “current” smokers (actively smoking or quit within the past 6 months). Household income was divided into quartiles (Q1–Q4), with higher quartiles representing higher income groups.

### 2.3. Study Parameters

The snoring (“Do you snore loudly?”), tiredness (“Do you often feel tired, fatigued, or sleepy during the daytime?”), observed apnea (“Has anyone observed you stop breathing during sleep?”), high BP (“Do you have high blood pressure?”), BMI, age, neck circumference, and male gender (STOP-Bang) questionnaire was used to screen for OSA [13]. The BMI cut-off value was set as 30 kg/m^2^ instead of 35 kg/m^2^ following criteria for Asian obesity classification in a previous study [14]. Each question was answered as “yes” and “no” and scored as 1 and 0, respectively. The total scores ranged from 0 to 8. A total score of 3 or more positive responses was regarded as high risk for OSA.

Health-related QoL in individuals with low and high risk of OSA was assessed using the 3-level EuroQoL 5-dimension component (EQ-5D-3L) [15,16]. The EQ-5D-3L is a widely recognized and reliable instrument for evaluating health statuses across various conditions. It consists of five dimensions: mobility, self-care, usual activities, pain/discomfort, and anxiety/depression. Respondents rated their health in each dimension at three levels: no problems, some problems, and extreme problems/unable to perform. The responses were used to calculate an index score ranging from 0 (a health state equivalent to death) to 1 (perfect health). The minimal clinically important difference (MCID) of EQ-5D-3L index score was 0.05 [17].

### 2.4. Statistical Analysis

To obtain accurate national estimates of the Korean adult population, the KNHANES used adjusted sampling weights for a complex sample design, involving techniques applied to different strata, such as primary sampling units and households. After applying these weights, statistical analysis was performed. Categorical variables were shown as frequencies with weighted percentages, while continuous variables were presented as means ± standard errors. Responses for each EQ-5D-3L dimension were compared between individuals at low and high risk for OSA using the Rao–Scott chi-square test.

The relationship between high and low risk for OSA and the EQ-5D-3L index score was analyzed using linear regression. Demographic and clinical factors related to the risk of OSA or the EQ-5D-3L index score were included in both univariate and multivariate linear regression analyses. A more negative value of a regression coefficient indicates a stronger negative impact on QoL. Determinants of each EQ-5D dimension were examined using logistic regression, with level 1 (no problems) being used as the reference. Levels 2 (some problems) and 3 (extreme problems) were combined as “some or extreme problems”. Statistical significance was determined with two-sided *p* values < 0.05. All analyses were conducted using SAS version 9.4 (SAS Institute Inc., Cary, NC, USA).

## 3. Results

### 3.1. Basline Characteristics

The eighth KNHANES recruited 22,559 individuals. Those under 40 years and those who had incomplete answers to the OSA and QoL questionnaires (*n* = 13,593) were excluded (Figure 1). Of the 8966 total participants, 6792 (75.8%) and 2174 (24.2%) were classified as having a low risk (STOP-Bang score ≤ 2) and high risk (STOP-Bang score ≥ 3) of OSA, respectively (Table 1). The mean age and proportion of men was significantly higher in the high risk OSA when compared with the low risk OSA group (age: 58.4 ± 0.3 years vs. 57.5 ± 0.3 years, *p* = 0.006; men: 83.8% vs. 36.1%, *p* < 0.001). The high OSA risk group had a greater proportion of former and current smokers (71.8% vs. 34.2%, *p* < 0.001) and a higher proportion of BMI ≥ 30 (14.7% vs. 2.0%, *p* < 0.001). Relevant comorbidities, such as asthma, COPD, hypertension, dyslipidemia, stroke, myocardial infarction or angina, and diabetes, were more prevalent in the high risk OSA group (all *p* < 0.05), whereas the household income was comparable between low-risk OSA and high-risk OSA group.

### 3.2. OSA and Health-Related QoL

**Figure 2:**
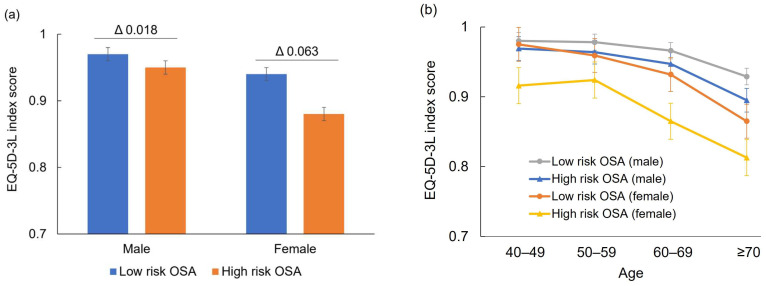
OSA and health-related QoL according to sex (**a**) and age (**b**). Delta (Δ) denotes mean difference of EQ-5D-3L score.

High risk OSA group showed significantly lower QoL scores when compared with low risk OSA group (0.939 ± 0.003 vs. 0.951 ± 0.002, *p* < 0.001). However, the mean difference was 0.012, which was within the MCID of the EQ-5D-3L. In the sex-based analysis, both male and female groups showed significant differences in the EQ-5D-3L scores between high risk OSA and low risk OSA group. However, the difference was more pronounced in the female (0.941 ± 0.002 vs. 0.878 ± 0.007, *p* < 0.001) than in the male (0.969 ± 0.002 vs. 0.951 ± 0.003, *p* < 0.001) (Figure 2a) groups. The female group only exceeded the MCID for EQ-5D-3L. Further analysis by age within sex subgroups indicated that the EQ-5D-3L scores was particularly lower among elderly individuals (aged ≥ 60 years) when compared to other age groups whether male or female. QoL scores were further decreased in individuals aged ≥ 70 years. Elderly females with a high risk of OSA showed the lowest QoL scores (Figure 2b).

### 3.3. Multivariate Analyses of the Relationship between OSA and the EQ-5D-3L Index Score

In the univariate linear regression analysis, the EQ-5D-3L index score was significantly associated with high risk OSA, with a regression coefficient of −0.012 (95% CI: −0.017–−0.006, *p* < 0.001). Several variables, including age, sex, current smoker, medical comorbidities, and household income, also showed significant relationships with the EQ-5D-3L index score (Table 2). In the multivariate analysis, the significance of the association between high risk OSA and the EQ-5D-3L index score was retained. The regression coefficient of high risk OSA in the multivariate model was −0.018 (95% CI: −0.025–−0.01, *p* < 0.001). Age, sex, current smoker, comorbidities including allergic rhinitis, stroke, myocardial infarction or angina, diabetes, and depression also showed significant associations with the EQ-5D-3L index score. Their regression coefficient was higher than that of a high risk of OSA.

### 3.4. OSA and EQ-5D Dimensions

In the analyses by each dimension, all five dimensions (mobility, self-care, usual activities, pain/discomfort, anxiety/depression) showed significantly different response rates between high risk OSA and low risk OSA group (Table S1). In the high risk OSA group, a larger proportion of individuals answered as having some or extreme problems when compared with low risk OSA group. Subgroup analyses by sex indicated that all five dimensions of the EQ-5D were still significantly impaired in both male and females with high risk OSA. However, the percentage of impairment was higher in the female group across all dimensions when compared to the male group.

We calculate the odds ratio (OR) by combining some and extreme problems as dependent variables (Table 3). Overall, individuals with high risk OSA exhibited significantly higher odds of experiencing problems in all dimensions when compared to those with low risk OSA. Notably, mobility issues were significantly associated with high risk OSA in females ≥65 years (OR = 1.825, 95% CI: 1.037–3.212, *p* = 0.037) and in males aged 40–64 (OR = 1.821, 95% CI: 1.011–3.281, *p* = 0.013). Similarly, self-care problems were more prevalent among those with high risk OSA, and this trend was particularly significant in females ≥65 years (OR = 2.445, 95% CI: 1.193–5.011, *p* = 0.015) and males aged 40–64 (OR = 3.757, 95% CI: 1.330–10.613, *p* = 0.013). Usual activities were significantly associated with high risk OSA only in males aged 40–64 (OR = 6.898, 95% CI: 2.864–16.615, *p* = 0.013). For the pain/discomfort and anxiety/depression domains, significant associations with high risk OSA were found in females ≥65 years and males ≥40 years. The impairment of all QoL domains was not associated with high risk OSA in females aged 40–64 years.

ORs were calculated from multivariate logistic regressions adjusted for age, sex, smoking, body mass index, household income, depression, asthma, COPD, dyslipidemia, angina/myocardial infarction, and stroke. Level 1 (no problems) was the reference. Levels 2 (some problems) and 3 (extreme problems) were combined as “some or extreme problems”.

## 4. Discussion

This population-based study found that females in the high risk OSA group had clinically lower QoL scores when compared to females in the low risk OSA group. QoL scores were most severely impaired in older females with high risk OSA. High risk OSA was significantly associated with lower QoL in a multivariate regression analysis after adjusting the relevant comorbidity variables. However, it was estimated that comorbidities had a greater impact on QoL than a high risk of OSA. The impact of OSA risk varied depending on the age, sex, and QoL domain.

The mean difference in the EQ-5D-3L index score was statistically different between the low and high risk OSA group. However, the mean difference was within the MCID. Therefore, our study results indicate that there was no clinically meaningful difference. Previous studies reported inconsistent results. Some studies reported impaired QoL in patients with OSA when compared to those without OSA [18,19,20], but other studies did not find significant differences in QoL between patients with and without OSA [21,22,23]. In a systematic review, a worse QoL score was noted in the OSA group when compared to the control group overall, and a significant association was not retained when study subjects were limited to studies with adjustment for covariates [10]. The Korean Genome and Epidemiology Study was conducted in a subset of the general population [24]. The association between OSA and QoL was marginally significant. However, this association disappeared after adjusting for covariates such as age, sex, education, BMI, drinking, smoking, hypertension, and diabetes [23]. This finding aligns with our study results, although different methods to assess QoL and define OSA were adopted. The regression coefficient for high risk OSA was 0.012, which was lower than other demographic factors and comorbidities in our study. Overall findings indicate that QoL in OSA should be assessed with adjustment for OSA-related covariates.

QoL scores were lower in older individuals and females, indicating a varied impact of OSA risk on QoL based on age and sex and highlighting the influence of comorbidities. Age is a significant risk factor for both OSA and comorbidities, with OSA further increasing the prevalence and severity of these conditions [25]. OSA manifests differently according to sex. It is more prevalent in men than in women, with an estimated ratio of 2:1 [25]. Males are at a higher risk of severe OSA compared to females [26]. Potential reasons for the higher prevalence in males include the effects of hormones on the muscle tone, the collapsibility of the upper airway, variations in body fat distribution between genders, and anatomical and functional differences in the pharynx [27]. However, post-menopausal females experience a significantly increased prevalence and severity of OSA compared to pre-menopausal females [26]. Research has shown that females tend to have an increased fat mass after menopause, with a greater concentration of fat in the upper body and trunk compared to the lower body, which makes them more susceptible to OSA [28]. Previous reports indicate that women with OSA are significantly more prone to experiencing anxiety and depression compared to men [29]. In our study, depression was the most significant factor associated with QoL. Therefore, age-related comorbidities, sex differences in OSA risk, and psychological conditions need to be considered in the assessment of QoL in individuals at high risk of OSA.

Dimension-specific analyses of QoL revealed that QoL was differently affected depending on age and sex. Older females had increased ORs for problems in all dimensions except usual activities, whereas younger females did not show significant impacts in any dimension. Conversely, younger males had increased ORs for problems in all dimensions, while older males did not experience significant impacts on mobility, self-care, and usual activities. The Australian study examined QoL in self-reported diagnosed OSA and high risk OSA [30]. A self-reported diagnosis of OSA was significantly associated with impairments across dimensions for both sexes, but only women with a diagnosis of OSA reported present feelings of anxiety or depression. However, this study did not examine the dimensional QoL according to the risk of OSA and age, limiting precise comparisons with our study. Many studies used the SF-36 questionnaire to assess QoL in OSA [10]. The OSA group showed worse QoL in at least one domain. Domains showing both statistically and clinically significant differences included physical functioning, physical role, pain, general health, vitality, emotional role, and mental health. The disease-specific questionnaire, the Calgary sleep apnea quality of life index (SAQLI), was applied in several studies [19,22,31]. All SAQLI domains were significantly worse in the OSA group than in the control group [19]. Studies had different characteristics of subjects and measurement questionnaires, which may lead to differing results in the dimensional approach to QoL. QoL in OSA was usually assessed using generic questionnaires such as SF-36. However, the correlation between SF-36 and the SAQLI was weak [31,32]. Generic questionnaires may not fully capture the important domains of QoL in OSA. Therefore, QoL should be measured using disease-specific instruments for OSA [10].

OSA affects QoL through several specific mechanisms. OSA leads to frequent interruptions in sleep due to arousals caused by airway obstruction. This fragmentation reduces the overall quality of sleep, resulting in daytime sleepiness and fatigue [33]. Episodes of apnea cause hypoxia. Chronic intermittent hypoxia can negatively impact cognitive function, mood, and overall physical health [33]. OSA is linked to an increased risk of hypertension, heart disease, and stroke. The stress on the cardiovascular system from repeated hypoxia and increased sympathetic nervous activity can severely affect overall health and QoL [34]. OSA is associated with a higher prevalence of depression and anxiety. The chronic sleep deprivation and hypoxia contribute to these mental health issues, further diminishing QoL [35]. OSA can impair cognitive functions such as attention, memory, and executive function, impacting daily activities and professional performance [1]. Chronic fatigue and excessive daytime sleepiness from poor sleep quality limit the ability to perform daily tasks and engage in social activities, reducing overall life satisfaction [33].

This study has several limitations that should be acknowledged. First, since the data pertain to the general Korean population, the majority of the participants in this study are Asian. This limitation may affect the generalizability of the findings to other ethnic groups. Second, the diagnosis of OSA was not confirmed through polysomnography, but was based on the STOP-Bang questionnaire, which may not provide the same accuracy as more rigorous methods. Third, OSA-specific instruments were not used to measure QoL. Therefore, our study may not have captured the specific impairment of QoL in the high risk OSA group. However, our study showed that generic QoL scores depended on age and sex, which can provide insights into the assessment of QoL in OSA. Fourth, the absence of a control group in this study complicates the direct attribution of QoL differences to OSA. Although comparisons are drawn between high risk and low risk groups, both groups are still within the OSA risk. Lastly, the cross-sectional design of this study limits the ability to establish causal relationships between OSA and QoL. Future longitudinal studies are needed to confirm this relationship.

## 5. Conclusions

This study demonstrated the relationship between OSA risk and QoL in a general adult population aged 40 years and older. Females in the high risk group showed clinically lower QoL when compared to those in the low risk group, while males did not show a relevant difference. Elderly females with high risk OSA exhibited the lowest QoL. The impact of comorbidities on QoL was greater than that of high risk OSA, highlighting the important role of OSA-related comorbidities and the need for their adjustment in the assessment of QoL in OSA. Generic questionnaires may not fully capture the important domains of QoL in OSA. Therefore, QoL should be measured using disease-specific instruments for OSA. These findings suggest that there is a considerable gap in the burden of OSA, indicating the need for a targeted approach to vulnerable individuals with OSA in the general population.

## Figures and Tables

**Table 1:** Baseline characteristics of study participants.

Characteristics	Total	Stop-Bang (≤2)	Stop-Bang (≥3)	*p*-Value
Age	57.8 ± 0.2	57.5 ± 0.3	58.4 ± 0.3	0.006
40~49	2098 (29.2)	1773 (32.4)	325 (19.7)	<0.001
50~59	2251 (30.1)	1570 (27.0)	681 (38.9)	
60~69	2259 (22.0)	1623 (20.7)	636 (25.8)	
over 70	2358 (18.8)	1826 (19.8)	532 (15.6)	
Sex (Male)	3890 (48.3)	2152 (36.1)	1738 (83.8)	<0.001
Smoking history				
Never	5255 (56.0)	4594 (65.8)	661 (28.1)	<0.001
Former	2210 (26.2)	1249 (19.7)	961 (44.6)	
Current	1355 (17.8)	819 (14.5)	536 (27.2)	
BMI				
<18.5 kg/m^2^	245 (2.7)	215 (3.2)	30 (1.2)	<0.001
18.5–24.9 kg/m^2^	5324 (59.7)	4424 (66.4)	900 (40.2)	
25.0–29.9 kg/m^2^	2817 (32.3)	1910 (28.3)	907 (43.8)	
≥30 kg/m^2^	459 (5.3)	150 (2.0)	309 (14.7)	
Medical condition				
Asthma	173 (1.7)	55 (0.7)	118 (4.4)	<0.001
COPD	30 (0.7)	12 (0.4)	18 (1.4)	0.006
Allergic rhinitis	778 (10.0)	593 (10.3)	185 (9.4)	0.320
Hypertension	2959 (29.2)	2049 (26.1)	910 (38.5)	<0.001
Dyslipidemia	2104 (21.6)	1485 (19.6)	619 (27.6)	<0.001
Stroke	201 (2.0)	133 (1.8)	68 (2.5)	0.046
Angina/MI	314 (3.2)	192 (2.6)	122 (4.6)	<0.001
Diabetes	1239 (12.4)	810 (10.5)	429 (17.9)	<0.001
Kidney disease	65 (0.6)	45 (0.6)	20 (0.7)	0.628
Depression	270 (2.7)	184 (2.6)	86 (3.2)	0.138
Household income bracket				
Low	2105 (18.9)	1605 (19.1)	500 (18.2)	0.408
Middle-low	2265 (24.3)	1727 (24.4)	538 (23.9)	
Middle-high	2203 (27.0)	1662 (27.2)	541 (26.5)	
High	2346 (29.8)	1759 (29.3)	587 (31.4)	

Continuous variables are shown as weighted means ± standard error (SE), while categorical variables are shown as unweighted frequency (weighted percentage). BMI, body mass index; COPD, chronic obstructive pulmonary disease; MI, myocardial infarct.

**Table 2:** Linear regression analysis of factors associated with the EQ-5D-3L index score.

Variables	Univariate Analysis		Multivariate Analysis	
	Coefficient	*p* Value	Coefficient	*p* Value
	B (95% CI)		B (95% CI)	
Age	−0.003 (−0.003–−0.003)	<0.001	−0.002 (−0.002–−0.001)	<0.001
Female (vs Male)	−0.025 (−0.030–−0.020)	<0.001	−0.031 (−0.039–−0.023)	<0.001
Smoking (%)				
Never	Ref.		Ref.	
Former	0.016 (0.011–0.021)	<0.001	−0.007 (−0.015–0.002)	0.123
Current	0.012 (0.005–0.019)	0.001	−0.01 (−0.02–−0.001)	0.037
BMI (%)				
<18.5 kg/m^2^	Ref.		Ref.	
18.5–24.9 kg/m^2^	0.018 (0.001–0.034)	0.035	0.027 (0.003–0.051)	0.029
25.0–29.9 kg/m^2^	0.014 (−0.003–0.031)	0.102	0.021 (−0.004–0.046)	0.096
≥30 kg/m^2^	−0.002 (−0.021–0.017)	0.839	0.015 (−0.014–0.043)	0.312
Medical condition				
Asthma	−0.042 (−0.062–−0.021)	<0.001	0.012 (−0.016–0.04)	0.392
COPD	−0.066 (−0.126–−0.007)	0.029	−0.055 (−0.11–0.001)	0.053
Allergic rhinitis	−0.003 (−0.011–0.004)	0.395	−0.015 (−0.026–−0.003)	0.011
Hypertension	−0.040 (−0.046–−0.034)	<0.001		
Dyslipidemia	−0.029 (−0.036–−0.023)	<0.001	−0.003 (−0.012–0.006)	0.470
Stroke	−0.091 (−0.121–−0.061)	<0.001	−0.041 (−0.077–−0.005)	0.026
Angina/MI	−0.047 (−0.062–−0.031)	<0.001	−0.024 (−0.044–−0.004)	0.018
Diabetes	−0.045 (−0.054–−0.036)	<0.001	−0.021 (−0.034–−0.009)	0.001
Kidney disease	−0.090 (−0.142–−0.038)	<0.001	−0.035 (−0.096–0.026)	0.265
Depression	−0.125 (−0.153–−0.097)	<0.001	−0.084 (−0.123–−0.045)	<0.001
STOP-Bang (≥3)	−0.012 (−0.017–−0.006)	<0.001	−0.018 (−0.025–−0.01)	<0.001
Household income				
Low	Ref.		Ref.	
Middle-low	0.062 (0.052–0.071)	<0.001	0.036 (0.024–0.048)	<0.001
Middle-high	0.081 (0.072–0.090)	<0.001	0.049 (0.037–0.061)	<0.001
High	0.090 (0.081–0.099)	<0.001	0.054 (0.043–0.064)	<0.001

BMI, body mass index; COPD, chronic obstructive pulmonary disease; MI, myocardial infarct.

**Table 3:** Association between STOP-Bang (≥3) and each EQ-5D dimension.

Variables	Total	Female (≥65 Years)	Male (≥65 Years)	Female (40–64 Years)	Male (40–64 Years)
	OR (95% CI)	OR (95% CI)	OR (95% CI)	OR (95% CI)	OR (95% CI)
Mobility					
Some or extreme problems	1.549 (1.203–1.994) **	1.825 (1.037–3.212) *	1.570 (0.979–2.517)	1.248 (0.679–2.293)	1.821 (1.011–3.281) *
Self-care					
Some or extreme problems	2.237 (1.433–3.492) **	2.445 (1.193–5.011) *	1.896 (0.90 –3.965)	2.305 (0.740–7.175)	3.757 (1.330–10.613) *
Usual activities					
Some or extreme problems	1.902 (1.358–2.664) **	1.540 (0.844–2.810)	1.280 (0.757–2.164)	1.629 (0.759–3.494)	6.898 (2.864–16.615) **
Pain/discomfort					
Some or extreme problems	1.760 (1.394–2.223) **	2.139 (1.234–3.708) *	1.613 (1.06 –2.443) *	1.178 (0.738–1.880)	2.147 (1.397–3.299) **
Anxiety/depression					
Some or extreme problems	1.970 (1.414–2.744) **	1.986 (1.010–3.908) *	2.775 (1.395–5.519) *	1.260 (0.646–2.455)	2.302 (1.157–4.580) *

* *p* < 0.05, ** *p* < 0.001.

## Data Availability

All data are available from the database of Korean National Health and Nutrition Examination Survey (KNHANES: https://knhanes.kdca.go.kr/knhanes/eng/index.do, accessed on 30 January 2024). The KNHANES allows access to all these data for any researcher who promises to follow the research ethics without approval and cost. Those seeking access to this articles’ data can download it from the website after promising to follow the research ethics.

## Supplementary Materials

The following supporting information can be downloaded at https://www.mdpi.com/article/10.3390/jcm13154360/s1, Table S1: Relationship between OSA risk and each EQ-5D dimensions.
