# Supplementary material for: Impact of High Risk of Obstructive Sleep Apnea on Health-Related Quality of Life: The Korean National Health and Nutrition Survey 2019–2021
Source: J Clin Med. 2024 Jul 25;13(15):4360. doi: 10.3390/jcm13154360 (PMC11313144; doi:10.3390/jcm13154360)
Supplement: Supplementary file 1 [file jcm-13-04360-s001.zip › Supplementary table.pdf]

Table S1. Relationship between OSA risk and each EQ-5D dimensions

Variables		Stop-Bang (≤ 2)	Stop-Bang (≥ 3)	P value
**Total (n, %)**				
Mobility				<0.001
	No problems	5096 (86.7)	1664 (83.1)	
	Some problems	971 (13.0)	451 (16.4)	
	Extreme problems	29 (0.4)	16 (0.4)	
Self-care				<0.001
	No problems	5854 (96.8)	1983 (94.9)	
	Some problems	225 (3.0)	141 (4.8)	
	Extreme problems	17 (0.2)	7 (0.3)	
Usual activities				<0.001
	No problems	5618 (93.6)	1882 (90.9)	
	Some problems	449 (6.1)	229 (8.5)	
	Extreme problems	28 (0.3)	20 (0.6)	
Pain/discomfort				0.006
	No problems	4652 (78.4)	1537 (74.8)	
	Some problems	1307 (19.9)	538 (23.3)	
	Extreme problems	136 (1.7)	56 (1.8)	
Anxiety/depression				0.014
	No problems	5531 (91.5)	1864 (89.3)	
	Some problems	535 (8.1)	251 (10.2)	
	Extreme problems	28 (0.4)	16 (0.5)	
**Female**				
Mobility				<0.001
	No problems	3429 (83.7)	261 (63.7)	
	Some problems	778 (15.9)	167 (35.2)	
	Extreme problems	23 (0.4)	8 (1.1)	
Self-care				<0.001
	No problems	4036 (96.1)	380 (89.3)	
	Some problems	180 (3.7)	55 (10.6)	
	Extreme problems	14 (0.2)	1 (0.2)	
Usual activities				<0.001
	No problems	3853 (92.2)	346 (82.3)	
	Some problems	356 (7.5)	82 (16.3)	
	Extreme problems	20 (0.3)	8 (1.3)	
Pain/discomfort				<0.001
	No problems	3064 (74.1)	223 (52.8)	
	Some problems	1052 (23.8)	184 (42.2)	
	Extreme problems	113 (2.1)	29 (5.0)	
Anxiety/depression				<0.001
	No problems	3762 (89.6)	335 (78.7)	
	Some problems	444 (9.9)	94 (19.9)	
	Extreme problems	22 (0.4)	7 (1.4)	
**Male**				
Mobility				<0.001
	No problems	1667 (92.2)	1403 (87)	
	Some problems	193 (7.5)	284 (12.7)	

	Extreme problems	6 (0.3)	8 (0.3)	
Self-care				<0.001
	No problems	1818 (98.2)	1603 (96)	
	Some problems	45 (1.8)	86 (3.7)	
	Extreme problems	3 (0.1)	6 (0.3)	
Usual activities				<0.001
	No problems	1765 (96.1)	1536 (92.6)	
	Some problems	93 (3.7)	147 (6.9)	
	Extreme problems	8 (0.2)	12 (0.5)	
Pain/discomfort				<0.001
	No problems	1588 (86.4)	1314 (79.2)	
	Some problems	255 (12.6)	354 (19.6)	
	Extreme problems	23 (1.1)	27 (1.2)	
Anxiety/depression				<0.001
	No problems	1769 (95)	1529 (91.4)	
	Some problems	91 (4.7)	157 (8.3)	
	Extreme problems	6 (0.3)	9 (0.3)	

Variables are shown as unweighted frequency (weighted percentage), and the p value was calculated by complex sample cross analysis (Rao-Scott chi-square test).
